# Multi-threshold image segmentation of 2D OTSU inland ships based on improved genetic algorithm

**DOI:** 10.1371/journal.pone.0290750

**Published:** 2023-08-25

**Authors:** Zhongbo Peng, Lumeng Wang, Liang Tong, Han Zou, Dan Liu, Chunyu Zhang

**Affiliations:** School of shipping and naval architecture, Chongqing Jiaotong University, Chongqing, China; Cyprus International University Faculty of Engineering: Uluslararasi Kibris Universitesi Muhendislik Fakultesi, TURKEY

## Abstract

Waterway transportation is a crucial mode of transportation, but ensuring navigational safety in waterways requires effective guidance of ships by the Water Resources Bureau. However, supervisors may only be interested in the ship portion of a complex image and need to quickly obtain relevant ship information. Therefore, this paper proposes a two-dimensional OTSU inland ships multi-threshold image segmentation algorithm based on the improved genetic algorithm. The improved algorithm enhances search accuracy and efficiency, improving image thresholding accuracy and reducing algorithm time complexity. Experimental verification shows the algorithm has excellent evaluation indexes and can achieve real-time segmentation of complex images. This method can not only address the challenges of complex inland navigation environments and difficult acquisition of target data sets, but also be applied to optimization problems in other fields by combining various metaheuristic algorithms.

## 1 Introduction

With the continuous development of science and technology, image processing area is getting more and more popular in science world. A picture often contains a large amount of information, but we are only interested in a local area or a specific area of the image, so image segmentation technology is extremely important. Image segmentation refers to the subdivision of a digital image into multiple non-overlapping sub-areas with special properties, and the features in the same sub-region have certain similarities, and the features of different sub-region show obvious differences. Common segmentation methods for image segmentation include threshold-based segmentation, region-based segmentation, edge-based segmentation, machine learning-based segmentation, level set-based segmentation, and active contour-based segmentation [1]. Among them, the threshold-based segmentation method is the most commonly used method for image segmentation, which is simple in calculation, high in stability and strong in operability. The core of image threshold segmentation is to select the optimal threshold. There are three classic methods for selecting the optimal threshold, the OTSU method [2], the maximum entropy method [3], and the minimum error method [4]. The OTSU method is aimed at single-threshold image segmentation, which can only divide the image into two types: background and target. In addition, the exhaustive search method needs a lot of computing time to find the optimal solution, and the efficiency is relatively low. Therefore, researchers have conducted extensive studies on the OTSU algorithm. Liu and Li [5] proposed a two-dimensional OTSU that expands the gray information system of the neighborhood. With the expansion of the pixel search space, the amount of calculation is also greatly increased. Sarkar [6] proposed a new optimization algorithm based on multi-level bee colony mating optimization technology. The experimental results of this algorithm show that the search speed can be effectively improved on the basis of the original algorithm. Huang [7] proposed an OTSU segmentation method based on fruit fly optimization, which simulates the fruit fly’s olfactory search for food to find the optimal threshold and improve both computational speed and segmentation performance. Li and Ding [8] presented an OTSU segmentation method based on sparrow optimization algorithm, which simulates sparrow’s foraging and anti-predator behavior to search for the optimal threshold. This algorithm showed significant improvements in optimization ability and iteration time. Horng MH [9] proposed a novel optimization algorithm based on multi-level bee colony mating optimization technique, and experimental results showed that this algorithm effectively improved the search speed on the basis of the original algorithm. Sun [10] optimized the OTSU method by combining the algorithm of satin bowerbird, and applied it to the segmentation of remote sensing images, which has advantages of good noise resistance and fast computational speed. Wu [11] proposed a cell segmentation method combining background subtraction and OTSU, which can objectively and accurately analyze the degree of cell convergence, and has high application value. Houssei [12] optimized OTSU by an improved golden jackal algorithm to solve the problem of multi-threshold, and applied it to skin cancer image segmentation in the medical field. This algorithm performed better than the original golden jackal optimization algorithm and achieved good results. Xing and Zhao [13] proposed an improved WOA algorithm (QGBWOA), introducing quasi-opposition based learning and Gaussian barebone for feature selection. The ability of QGBWOA to solve practical problems was validated through feature selection and multi-threshold image segmentation applications. Han and Chen [14] proposed the RDMVO algorithm, which is the multi-verse optimizer algorithm using the Rosenbrock method and diffusion mechanism. This algorithm is primarily used for multilevel threshold image segmentation from COVID-19 chest X-ray images. Experimental results have demonstrated that RDMVO exhibits strong competitiveness in benchmark functions and image segmentation experiments compared to other metaheuristic algorithms. Wang and Li [15] proposed a two-stage medical image segmentation method that includes image fusion and image segmentation. Experimental results demonstrate that this algorithm outperforms seven other algorithms in terms of subjective visual evaluation and objective performance metrics. Chen and Cai [16] proposed a specialized multi-threshold image segmentation method for images of Lupus Nephritis. The research indicates that this algorithm is an effective approach for segmenting renal pathology images. Wang [17] proposed a new multilevel thresholding method that uses cooperative pigeon-inspired optimization algorithm with dynamic distance threshold. This algorithm not only achieves higher quality segmentation results but also exhibits improved stability. Mohamed [18] proposed a novel Moth Swarm Algorithm. The algorithm introduces the mechanisms of associative learning, adaptive Gaussian walks, and spiral motion. Experimental results indicate that this algorithm is suitable for solving non-smooth and complex problems, showing some potential in multi-objective optimization problems. Wang [19] proposed a new nature-inspired metaheuristic algorithm called Monarch Butterfly Optimization (MBO). This algorithm is simple and fast, and it is used for continuous optimization problems, capable of finding better function values on most benchmark problems. Li and Chen [20] proposed a new stochastic optimizer called Slime Mold Algorithm (SMA). This algorithm adopts a unique mathematical model, which ensures both exploratory performance and superior exploitative performance. It is suitable for practical engineering optimization problems. Ahmadianfar [21] proposed an efficient optimization algorithm based on the Runge Kutta method. This algorithm demonstrates good exploitative and exploratory capabilities on both unimodal and multimodal test functions, exhibiting better performance in optimizing complex real-world problems. Tu and Chen [22] proposed a new stochastic optimizer called the Colony Predation Algorithm (CPA), based by the corporate predation of animals in nature. This algorithm achieves a proper balance between exploitation and exploration and can quickly converge in the early and middle stages without falling into local optimization. Su and Zhao [23] proposed an efficient optimization algorithm based on the physical phenomenon of rime-ice, called RIME. This algorithm introduces a soft-rime search strategy and a hard-rime puncture mechanism. Experimental results have demonstrated the effectiveness of this algorithm in solving global optimization problems. Ren [24] proposed a multi-level thresholding segmentation method based on modified differential evolution, which has high convergence accuracy and the ability to escape from local optima. It performs well in segmenting pathological images and can produce high-quality segmentation results. Emam [25] proposed a modified version of the reptile search algorithm, aimed at addressing threshold selection problems for global optimization and multi-level image segmentation. Compared to other algorithms, this algorithm has stronger search capabilities and has demonstrated good optimization performance and segmentation results in practical applications. Hosny [26] proposed a multilevel thresholding technique that uses OTSU and Kapur’s entropy methods as fitness functions to determine the optimal threshold values. This technique has shown outstanding performance in the field of 2D and 3D medical image segmentation, improving the efficiency of the algorithm. Zhu [27] proposed an efficient multi-threshold image segmentation method using boosting whale optimizer, which introduces the Levy operator and chaotic random mutation strategy. This technique significantly enhances the algorithm’s optimization ability and convergence speed and has shown excellent performance in the field of skin cancer image segmentation. Zheng [28] proposed an improved particle swarm optimization algorithm, introducing a new elite particle search strategy. This algorithm achieves higher segmentation accuracy and efficiency and has excellent development and exploration capabilities.

Among which, the two-dimensional OTSU algorithm makes full use of the spatial location information of image pixels and pixel neighborhoods, so it has greater noise immunity than the one-dimensional OTSU algorithm that only uses the image gray histogram [29]. But the two-dimensional OTSU method increases the computational complexity [30]. In order to overcome the shortcomings of the two-dimensional OTSU method, such as high computational complexity, poor real-time performance, and susceptibility to noise, this paper proposes a two-dimensional OTSU inland ships multi-threshold image segmentation based on an improved genetic algorithm [31]. This method first preprocesses the image, then analyzes the two-dimensional histogram of the image. Finally, the five algorithms are compared and analyzed using evaluation metrics such as PSNR and time. For the multi-threshold image segmentation problem in this paper, genetic algorithm, as a global optimization method, can effectively search for the optimal combination of multiple thresholds to achieve accurate image segmentation. In addition, it is also adaptive and flexible, and can be adjusted and optimized according to the characteristics and requirements of the problem. For different types of images and segmentation tasks, the parameters and fitness function of the genetic algorithm can be adjusted to obtain optimal multi-threshold segmentation results. The improved algorithm enhances search accuracy and efficiency, improving image thresholding accuracy and reducing algorithm time complexity. Experimental verification shows the algorithm has excellent evaluation indexes and can achieve real-time segmentation of complex images. Combining the characteristics of genetic algorithms, which are less prone to being trapped in local optima and easy to parallelize, improvements have been made to genetic algorithms and applied to two-dimensional OTSU multi-threshold image segmentation. This approach enables the discovery of the best set of thresholds with less time, achieving good segmentation results.

## 2 OTSU algorithm

### 2.1 One-dimensional OTSU

OTSU is further derived based on the two-dimensional inter-class variance method and the least squares method. Its basic idea is: set the segmentation threshold, divide the image into two parts according to the gray level, the target and the background, and traverse all the gray values, find the threshold that maximizes the variance between classes and minimizes the variance within a class. At this time, the threshold T is the optimal image segmentation threshold.

Let *A* be an image with a gray level of *L*, the total number of pixels is *N*, and the i -level pixels are *N*_*i*_, and *P*_*i*_ represents the probability of occurrence of the *i*-level pixel. *P*_*i*_ is calculated as Eq (1).


$$P_{i}=\frac{N_{i}}{N},i=0,1,2\ldots ,L-1$$ (1)

Select the threshold *K*, and divide the image into *C*_0_ (target) and *C*_1_ (background) according to the threshold *K*, where the pixel gray level of the target class is [0~*K*], and the pixel gray level of the background class is [*K*+1~*L*].

The total average gray level of image *A* is *μ*, the average gray level of the target class is *μ*_0_ and the background class is *μ*_1_, specifically expressed as Eqs (2), (3) and (4):

$$\mu_{0}=\sum_{i=0}^{K}\frac{iP_{i}}{\omega_{0}}$$ (2)

$$\mu_{1}=\sum_{i=K+1}^{L-1}\frac{iP_{i}}{\omega_{1}}$$ (3)

$$\mu =\omega_{0}\mu_{0}+\omega_{1}\mu_{1}$$ (4)

The between-class variance is defined as Eq (5):

$$\sigma_{B}{}^{2}=\omega_{0}{\left(\mu_{0}-\mu \right)}^{2}+\omega_{1}{\left(\mu_{1}-\mu \right)}^{2}$$ (5)

In the formula: $\omega_{0}=\sum_{i=1}^{n}P_{i},\omega_{1}=1-\omega_{0}$. The threshold K traverses all the gray values, and calculates the inter-class variance under different *K* values, so that the *K* value with the largest inter-class variance is the optimal threshold.

### 2.2 Two-dimensional OTSU multi- threshold algorithm

Two-dimensional OTSU is a binary group composed of image gray value and neighborhood average gray value. The two-dimensional OTSU method judges the boundary information by comparing the difference between the gray value of the current pixel and the average gray value of *a*×*a* pixels in the neighborhood [32]. Let *F*(*x*,*y*) be a grayscale image with a grayscale level of L and a resolution of *M*×*N*, and corresponding to each pixel, take the gray value of *a*×*a* pixels in the neighborhood to obtain a smooth image *f*(*x*,*y*) with the same gray level as the original image.

**Fig 1:**
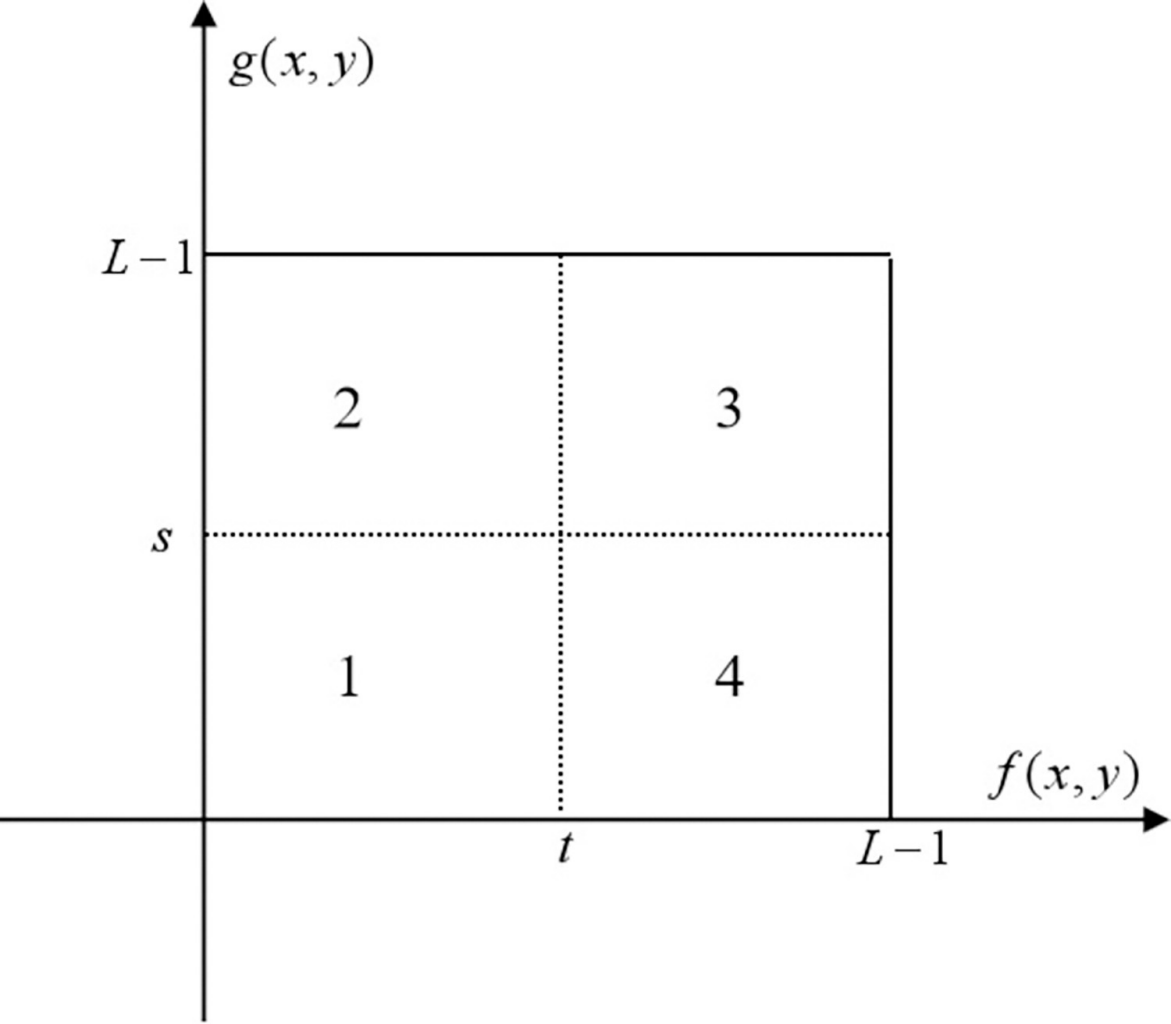
Two-dimensional histogram with partitioned regions.

For two-dimensional OTSU single threshold segmentation, the threshold (*s*,*t*) divides the two-dimensional histogram into 4 parts. The 1 and 3 areas adjacent to the diagonal are the target and background, and the 2 and 4 areas are edges and noise. Since there are fewer pixels on the diagonal in the 2 and 4 areas, the pixels are mainly concentrated near the diagonal, so the probability of the 2 and 4 areas is approximately 0. As shown in Fig 1. In the 1 and 3 area, use the point gray value-neighborhood gray value mean, take the point gray value as the abscissa, and the neighborhood gray value as the ordinate.

**Fig 2:**
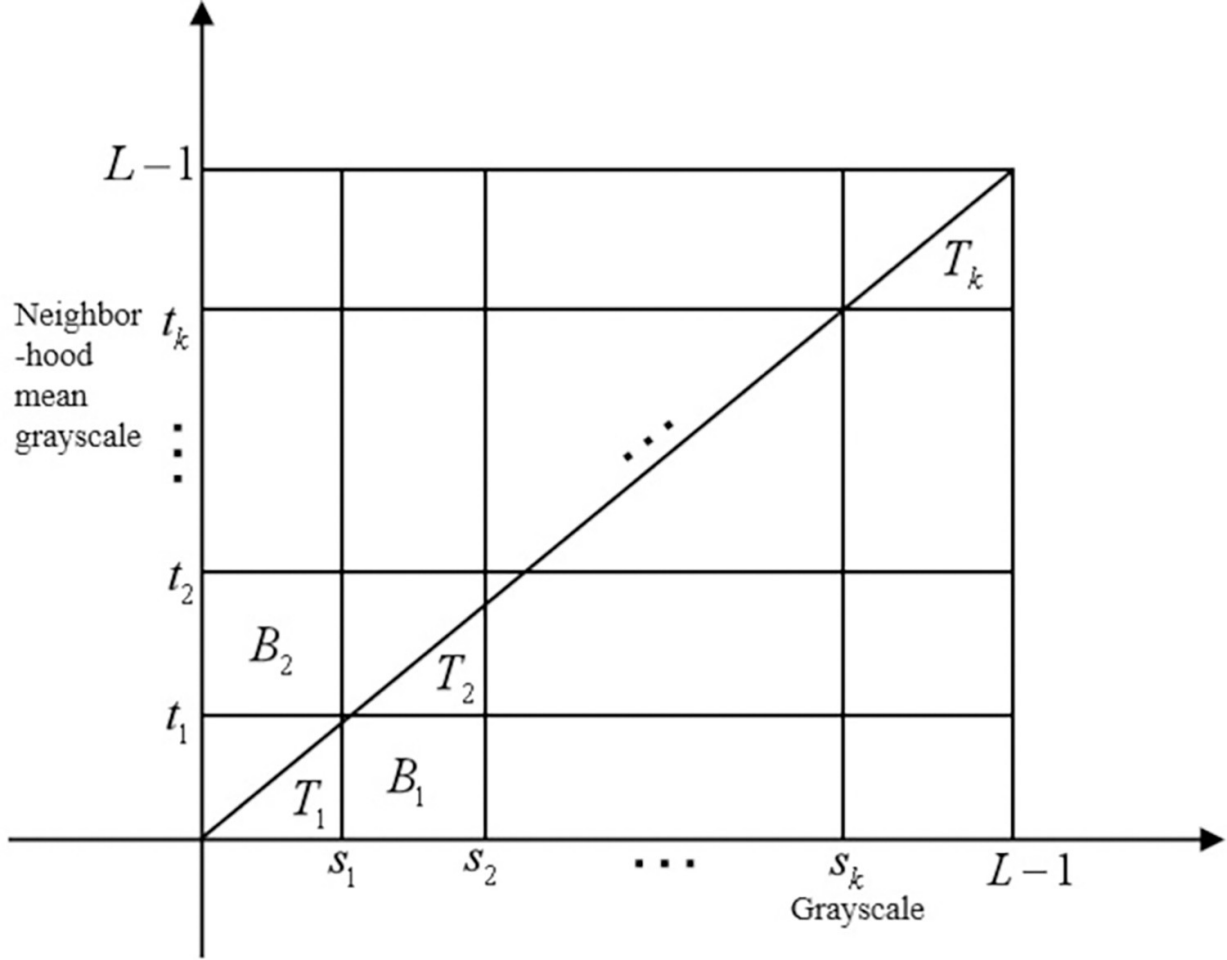
Two-dimensional OTSU *k* threshold segmentation histogram.

The two-dimensional OTSU *k* threshold histogram is shown in Fig 2. Extend the segmentation threshold gray level to (*s*_*k*_,*t*_*k*_), where *k* represents the number of segmentation thresholds. Assuming that *T*_1_ represents the background area and *T*_2_…*T*_*k*_ represents the target area, the probability calculation formula of the background area is Eq (6):

$$\omega_{0}=\sum_{i=0}^{s_{1}}\sum_{j=0}^{t_{1}}P_{ij}$$ (6)

The probability calculation formula of the target area T _k_ is Eq (7):

$$\omega_{k}=\sum_{i=s_{2}}^{s_{k}}\sum_{j=t_{2}}^{t_{k}}P_{ij},k\geq 2$$ (7)

In the formula, $Pij=\frac{g_{ij}}{M\times N}$ represents the joint probability density function of the pixel point (*x*,*y*) whose gray level is *i* and the average gray level is *j* in the neighborhood, and *g*_*ij*_ represents the number of pixels.

The formula for calculating the mean value vector of the background region is Eq (8):

$$u_{1}={\left(u_{1i},u_{1j}\right)}^{T}$$ (8)

The formula to calculate the mean vector of *T*_*k*_ in the target region is Eq (9):

$$u_{k}={\left(u_{ki},u_{kj}\right)}^{T}={\left(\frac{1}{\omega_{k}}\sum_{i=s_{2}}^{s_{k}}\sum_{i=t_{2}}^{t_{k}}iPij,\frac{1}{\omega_{k}}\sum_{i=s_{2}}^{s_{k}}\sum_{i=t_{2}}^{t_{k}}jP_{ij}^{}\right)}^{T}$$ (9)

Finally, the segmentation threshold is defined as (*s*_1_,*t*_1_),(*s*_2_,*t*_2_),…(*s*_*k*_,*t*_*k*_), and the two-dimensional OTSU multi-target segmentation distance measure function is expressed as Eq (10):

$$R(s_{1},t_{1},s_{2},t_{2},\ldots s_{k},t_{k})=\sum_{n=1}^{k}\omega_{n}[{\left(u_{ni}-ui_{L}\right]}^{2}+\omega_{n}[{\left(u_{nj}-u_{jL}\right)}^{2}]$$ (10)

When the distance measure function *R*(*s*_1_,*t*_1_,*s*_2_,*t*_2_,…*s*_*k*_,*t*_*k*_) obtains the maximum value, the optimal threshold $\left(s_{1}^{*},t_{1}^{*}),(s_{2}^{*},t_{2}^{*}),\ldots (s_{k}^{*},t_{k}^{*}\right)$ for two-dimensional OTSU multi-threshold segmentation can be obtained, and then the objective function is optimized through the improved genetic algorithm to find the best set of thresholds.

## 3 Two-dimensional OTSU based on improved genetic algorithm

### 3.1 Introduction to genetic algorithm

Genetic Algorithm was first proposed by Professor Holland in the United States, and it is a computational model that simulates natural selection and genetic evolution of organisms in nature. Genetic algorithm is one of the key technologies of modern intelligent computing. It is widely used in machine learning, software technology, pattern recognition, signal processing, adaptive control and other fields. The basic idea is to find the optimal solution [33, 34]. The process of finding the threshold in this paper is essentially the process of finding the optimal solution, which conforms to the basic idea of genetic algorithm. In order to shorten the optimization time, the genetic algorithm is applied in the process of finding the threshold value, so as to realize the optimization process.

### 3.2 Two-dimensional OTSU algorithm based on improved genetic algorithm

By combining the genetic algorithm with the OTSU algorithm, the optimal threshold for image segmentation can be obtained faster. However, the traditional genetic algorithm has the problems of early convergence and easy to fall into local optimal solution, which will lead to poor segmentation results. The setting of genetic algorithm control parameters has a great influence on the running time and final result of the algorithm. The number of targets in the picture is different, the required optimization parameter *k* is also different, and the complexity of the algorithm is also different [35, 36]. In order to avoid such problems, this paper makes improvements to the traditional genetic factors and fitness functions. The improved genetic algorithm process is as follows:

(1) Coding. Genetic algorithm expresses all possible solutions with a certain code, which is called chromosome genotype. As the first step of the genetic algorithm, the efficiency of the algorithm has a lot to do with it, so coding is very important. In this paper, the objective of optimization is the segmentation threshold *K* being [0~255]. Therefore, according to the different classification numbers s, each chromosome as an 8×(*s*−1)-bit binary code, and each 8-bit represents a *K* value [37].
(2) Generation of the initial population. In the process of population initialization, individuals are generally randomly generated. The solution space of the problem is usually uniformly sampled, and a certain number of individuals are randomly generated, from which good individuals are selected to form the initial population. In this study, 20 individuals were randomly generated with equal probability between 0 and 255 as the initial population for the first optimization search.
(3) Choose the fitness function. Fitness is the deterministic criterion for the selection of individual survival opportunities in the group, and it is unique, so the evolutionary behavior of the population is directly determined by the fitness function. In each generation, there are many different chromosomes, and the key to deciding which chromosomes to pass on to the next generation is the fitness value of the individual. The larger the fitness value, the more likely it is to approach the optimal solution. In this paper, we choose the distance measure function *R*(*s*_1_,*t*_1_,*s*_2_,*t*_2_,…*s*_*k*_,*t*_*k*_) is the fitness function.
(4) Determination of genetic operators. Genetic operators include selection, crossover, and mutation, which simulate the specific process of biological evolution in nature and are the core of genetic algorithms with powerful search capabilities.

1. Selection is the process of selecting chromosomes from the population to generate a new population. The higher the fitness value of a chromosome, the greater the probability of being selected. There are many selection methods, such as Roulette wheel selection, Boltzmann selection, ranking selection, league selection, elite selection, etc. [38]. Elitist selection directly preserves the individuals with the highest fitness values for the next generation, which may overlook potential excellent solutions in other individuals, hinder population diversity, and limit the algorithm’s exploration capability. In this paper, the roulette wheel selection is used. This strategy first assigns probability according to the fitness ranking of individuals in the group, and then performs roulette selection according to this probability. The selection process is stochastic, where individuals with higher fitness values have a greater probability of being chosen, but individuals with lower fitness values still have a chance of being selected. This provides some room for survival for individuals with lower fitness values, contributing to global exploration in the search space, maintaining population diversity, and increasing the algorithm’s exploration capability.
2. Crossover and mutation

Crossover is the process of imitating the genetic recombination of biological reproduction in nature, passing on excellent genes to the next generation, and generating new individuals with more excellent genetic structures. Variation is the phenomenon that the genes on the chromosomes undergo gene mutations during the evolution process of organisms in the imitation of the biological world, and the mutations will change the structure and characteristics of the chromosomes.

When calculating the optimal threshold by genetic algorithm, the selection of crossover rate and mutation rate is very important [39]. The adaptive basic genetic algorithm proposed by Javadi and Aminian can automatically adjust the crossover rate and mutation rate according to the fitness [40]. However, when the individual fitness is infinitely close to the maximum fitness, the crossover probability and mutation probability are small; when the individual fitness is greater than or equal to the maximum value, the crossover rate and mutation rate are close to 0, which can easily lead to local optimum. In order to avoid this situation, the algorithm can be further improved so that the algorithm can obtain the global optimal solution. The improved crossover rate and mutation rate formula are shown in Eqs (11) and (12).


$$p_{c}=\{\begin{array}{l} p_{cm},f'<f_{a}\\ p_{cm}.e^{\frac{f'-f_{a}}{f_{m}-f_{a}}},f'\geq f_{a} \end{array}$$ (11)

$$p_{m}=\{\begin{array}{l} p_{mm},f<f_{a}\\ p_{mm}.e^{\frac{f-f_{a}}{f_{m}-f_{a}}},f\geq f_{a} \end{array}$$ (12)

Where, *f*_*m*_ is the maximum fitness value of the population, *f*_*a*_ is the average fitness value of the population in each generation, *f*’ is the larger fitness value of the two individuals to be crossed, *f* is the fitness of the individuals to be changed, *p*_*cm*_ is the maximum crossover probability, *p*_*mm*_ is the maximum mutation probability.

**Fig 3:**
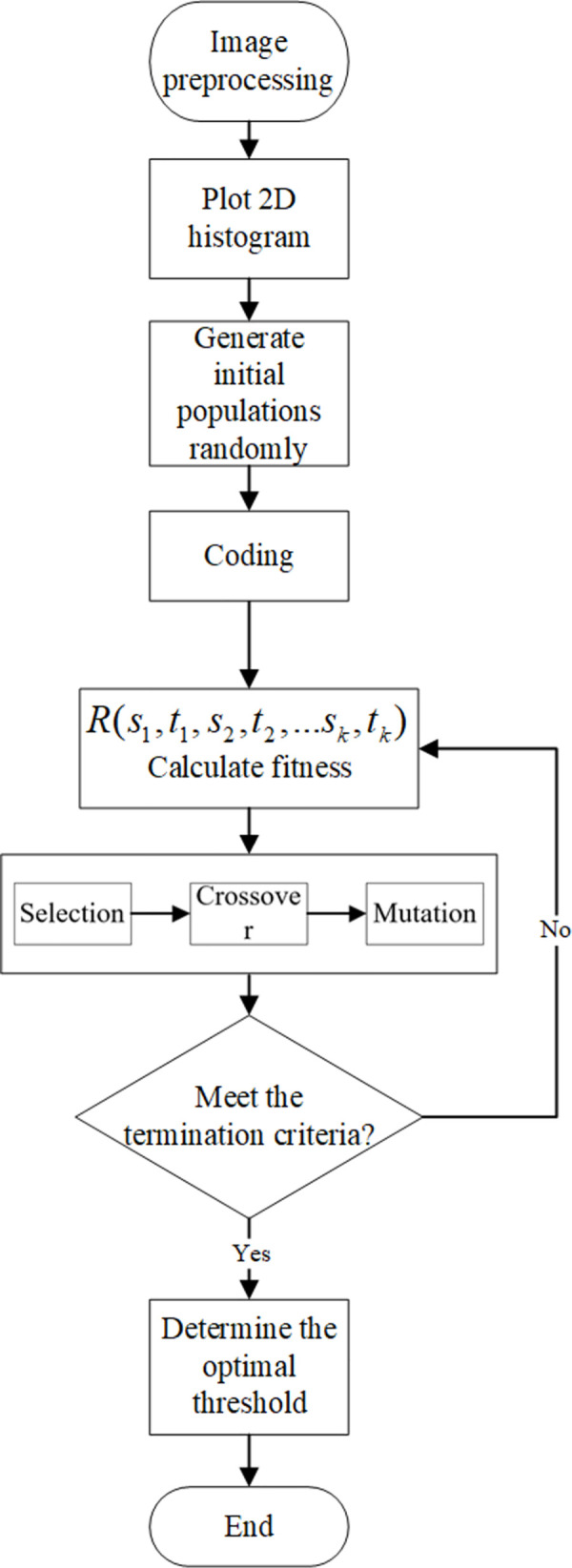
Image segmentation flow chart.

(5) Termination criterion setting. The maximum number of evolutionary generations is 40, and the termination criterion is to stop the iteration when the difference between the average fitness values of two adjacent generations of individuals is within [0,0.005]. If the criterion of termination is not satisfied, take the new group as the initial group, go to step (3), and output the result if it is satisfied.
(6) Determination of optimal threshold. The one with the greatest fitness in the last generation is the best result, and it is decoded to find the best segmentation threshold. The specific flow chart of this paper is shown in Fig 3.

## 4. Experiment and result analysis

In order to verify the effectiveness and accuracy of the above method, this paper uses the Python programming language to implement the two-dimensional OTSU multi-threshold algorithm of the improved genetic algorithm to segment the ship images. By comparing and analyzing the performance indicators of iterative method, bimodal method, one-dimensional OTSU algorithm and two-dimensional OTSU algorithm, the robustness and reliability of the algorithm in this paper are highlighted.

### 4.1 Image preprocessing

**Fig 4:**
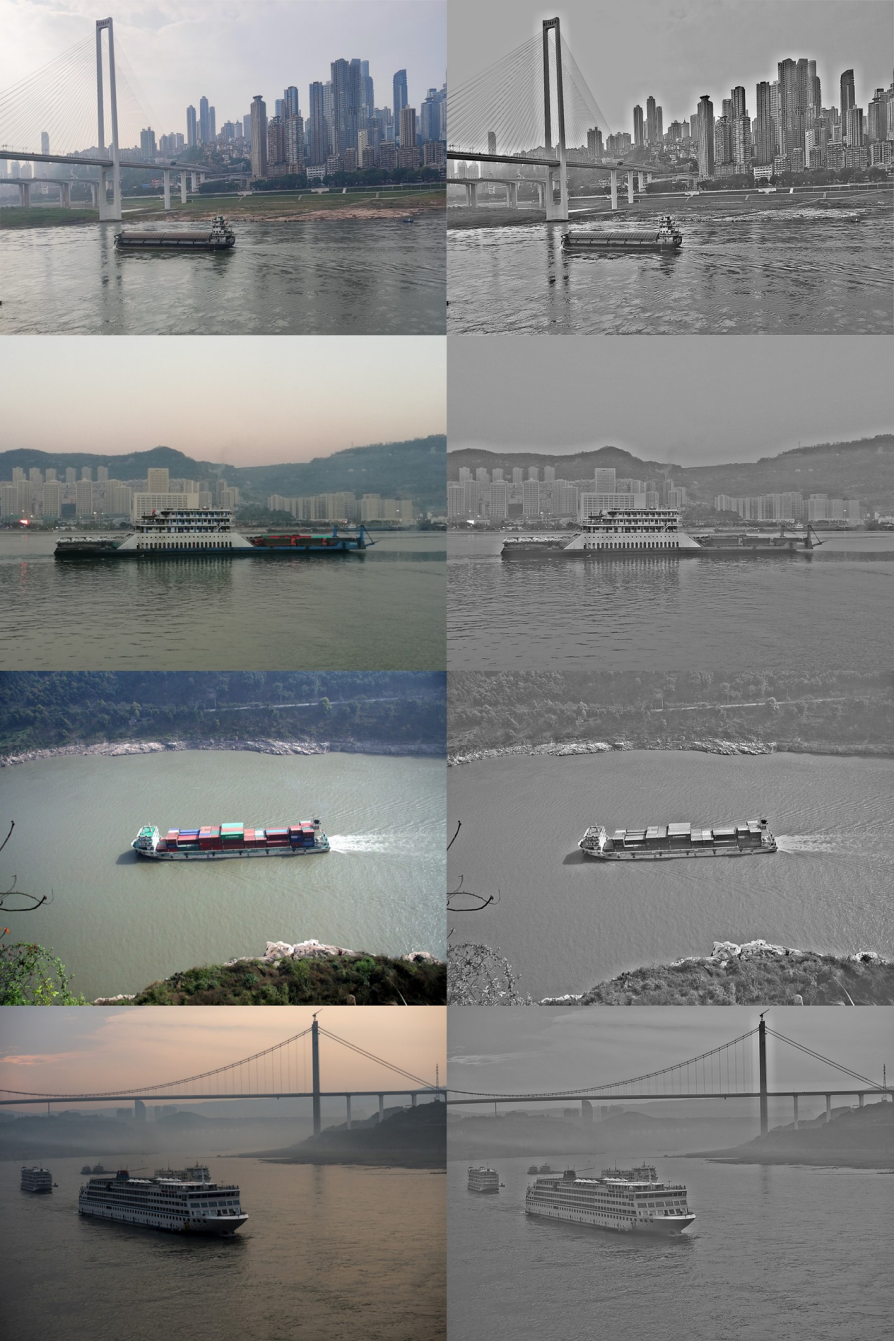
Original image (left) and preprocessing (right).

The images used in this paper were collected on site by ourselves. The preprocessed image is shown in Fig 4.

### 4.2 Comparative experiment

**Fig 5:**
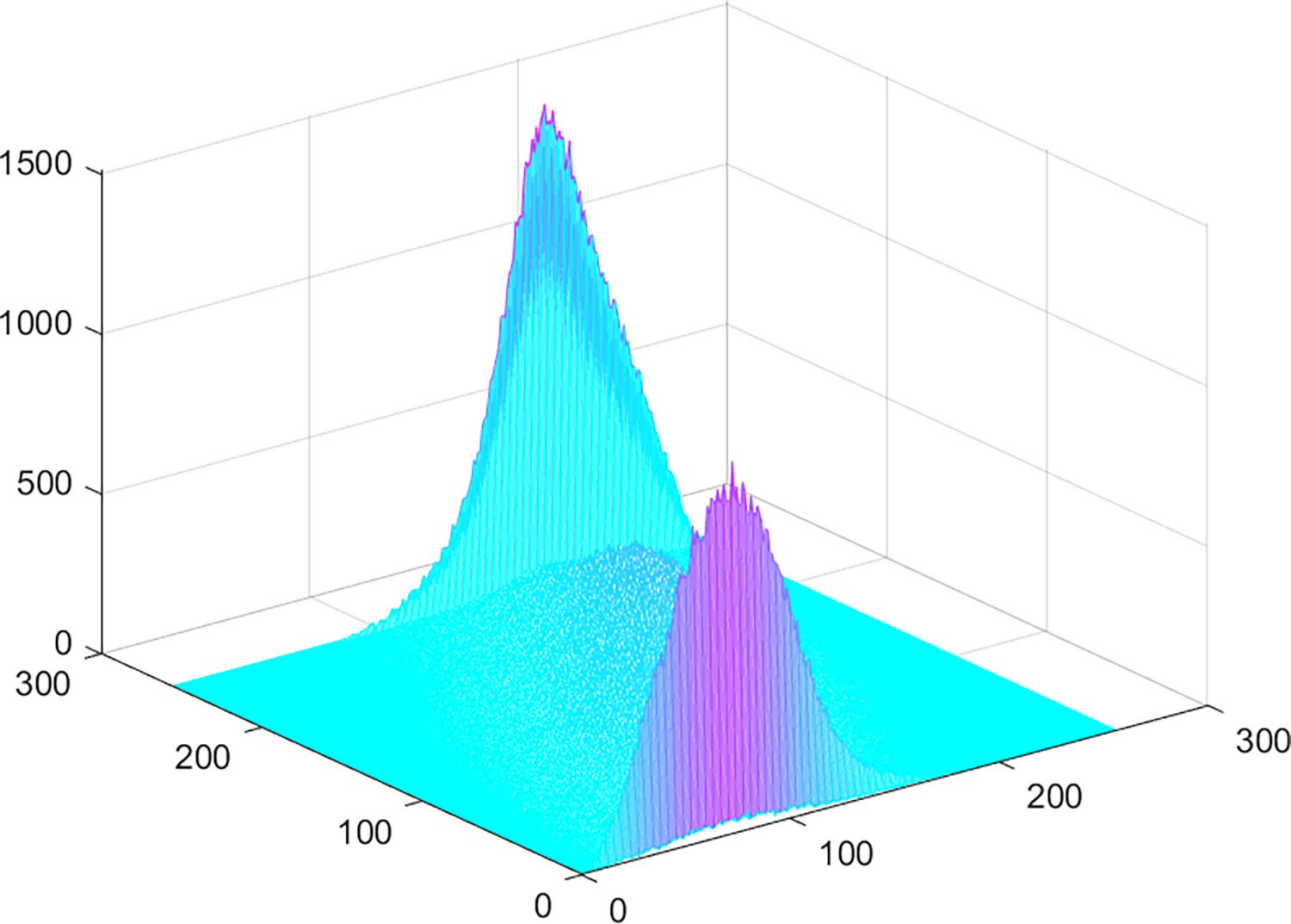
Two-dimensional histogram.

The histogram of the two-dimensional OTSU algorithm for the image is shown in Fig 5. It can be seen that the vicinity of the diagonal of the plane is the main division of the probability peak corresponding to the image midpoint grayscale-regional grayscale mean, and it generally presents a state of double peaks and a valley. This is mainly because among all the pixels in the image, the proportion of the background point and the target point is optimal, and in the target area and the background area, the pixel gray level distribution is very uniform, and the gray level of each point and its area The difference in the mean value of the inner gray level is very small. Therefore, in the two-dimensional histogram of the image, most of the points are distributed near the diagonal, and the target and the background are represented by two peaks. At places far from the diagonal, the height of the peaks decreases rapidly, and these points reflect noise points, stray points and edge points in the image.

**Fig 6:**
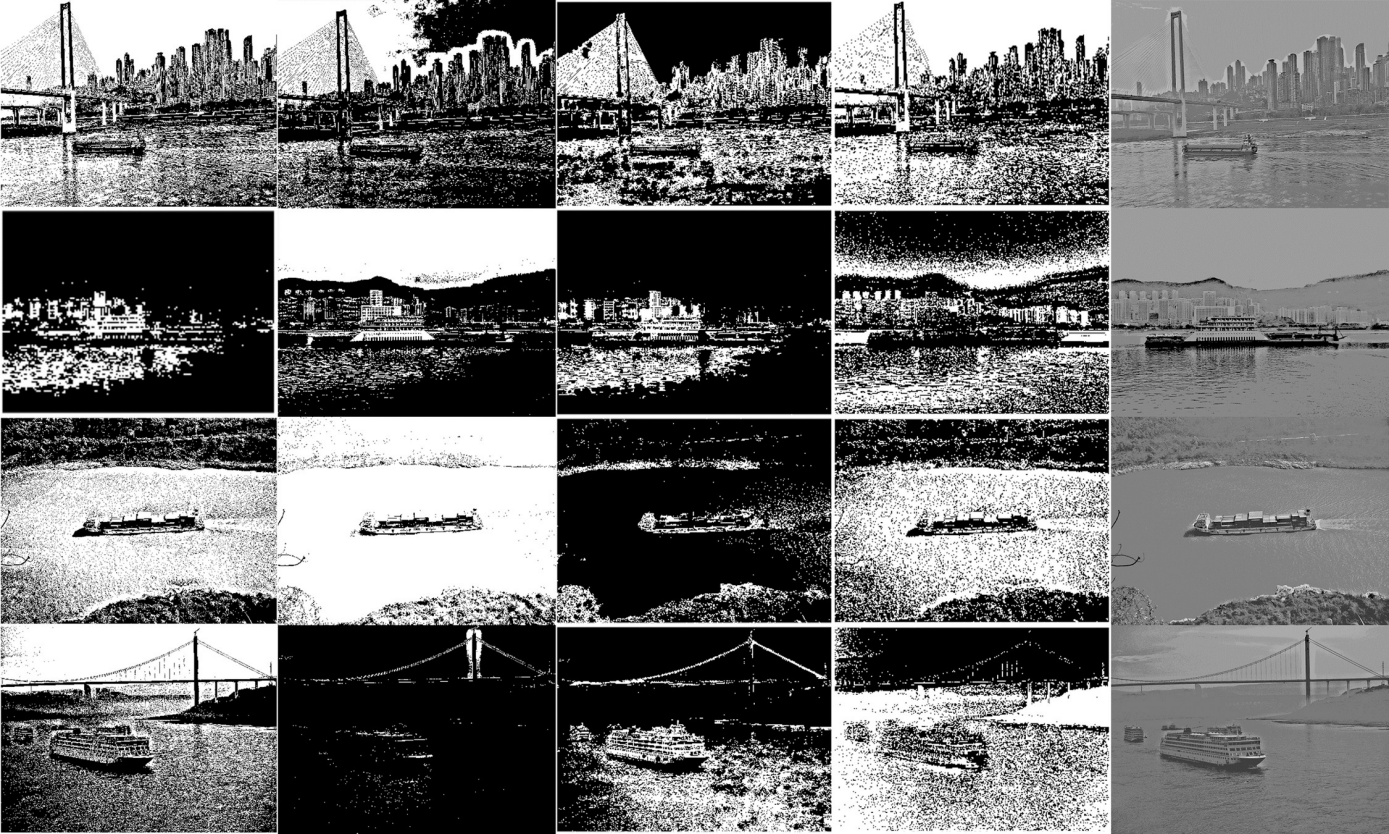
Algorithm effect comparison chart.

In this study, the iterative method, bimodal method, one-dimensional OTSU algorithm, two-dimensional OTSU algorithm and the algorithm in this paper are used for comparative experiments. The segmentation results of each algorithm are shown in Fig 6. Fig 6 is composed of 20 small images arranged in four rows. The first row shows the results of five processing methods for Image 1, from left to right: iterative method, bimodal method, one-dimensional OTSU, two-dimensional OTSU, and the method proposed in this paper. The second row shows the results of the five processing methods for Image 2, and so on for the third and fourth rows.

It can be seen from the above figure that the effect of one-dimensional OTSU is better than that of iterative method and bimodal method, and the processing effect of the algorithm in this paper is better than that of the previous algorithms. Using the two-dimensional OTSU algorithm, while considering the spatial neighborhood pixels, the genetic algorithm is used to optimize the time complexity, so that it achieves a better segmentation effect. Regarding the segmentation of the bridge in Image 1, only the algorithm presented in this paper can accurately show the shape of the bridge and effectively separate the distant buildings from the sky. Furthermore, in Image 4, there is a small vessel on the left side of the large target hull, and the algorithm described in this paper can clearly differentiate between these two target ships on the water surface. The segmentation thresholds of the proposed method are listed in Table 1, which consist of 5 two-dimensional arrays. The selection of these arrays has been verified in detail through experiments to ensure good segmentation results in the selected images. Using these thresholds, the algorithm proposed in this paper can clearly segment the targets in the image, presenting a clear and distinct effect with improved precision and accuracy in image segmentation. It can be seen that the segmentation results of the other four algorithms are slightly inferior to the proposed algorithm, indicating that the proposed algorithm has certain advantages in image segmentation.

**Table 1:** Segmentation thresholds of different algorithms.

Image	Iterative method	Bimodal method	One-dimensional OTSU	Two-dimensional OTSU	Algorithm in this paper
1	99	20,153	102	(91,117)	**(27,32) (63,69) (92,99) (142,153) (219,225)**
2	102	53,121	115	(102,123.5)	**(48,51) (70,74) (114,122) (185,191) (205,212)**
3	123	89,142	130	(127,232)	**(43,47) (72,79) (99,101) (154,167) (201,214)**
4	114	31,117	109	(101,212)	**(31,40) (99,103) (135,142) (185,187) (221,230)**

### 4.3 Segmentation result evaluation

In order to verify the segmentation effect of different algorithms, this paper uses peak signal-to-noise ratio (PSNR) and segmentation time to evaluate each algorithm. PSNR is a commonly used index to measure image quality. The error between the processed image and the original image pixel is calculated point by point to determine the image quality score. The calculation formula of PSNR is shown in Eqs (13) and (14).


$$MSE=\frac{1}{mn}\sum_{i=1}^{n}\sum_{i=1}^{n}\|I(i,j)-K(i,j){\|}^{2}$$ (13)

$$PSNR=10\;\log_{10}(\frac{MAX_{I}{}^{2}}{MSE})$$ (14)

Among them, *I* is the processed image with the size of *m*×*n*, *K* is the original image with the size of *m*×*n*, MSE is the mean square error, MAX_I_ is the maximum possible pixel value of the picture. The larger the PSNR value, the better the quality of the segmented image and the smaller the distortion of the image [41]. The PSNR values of different algorithms are shown in Table 2.

**Table 2:** PSNR values of each algorithm.

Image	Iterative method	Bimodal method	One-dimensional OTSU	Two-dimensional OTSU	Algorithm in this paper
1	7.7752	7.2028	6.2635	7.9467	**20.3890**
2	6.8453	7.3762	5.9527	7.0556	**19.6626**
3	7.7771	6.4539	6.3461	7.9406	**20.3527**
4	7.8532	6.3617	6.5943	6.4334	**22.4794**

Based on the experimental results, Table 2 shows that there is not much difference in the PSNR values and segmentation performance between the iterative method and the bimodal method. However, from Fig 6, we can see that the iterative method performs better than the bimodal method for Image 4, indicating that the performance of the algorithm may vary for different images. Overall, the segmentation effect of two-dimensional OTSU is higher than the average PSNR value of one-dimensional OTSU by 1.0549, demonstrating a certain degree of improvement. The algorithm proposed in this paper shows high robustness, with an overall PSNR value that is 13.3768 higher than the average of the two-dimensional OTSU algorithm, and the best performance on Image 4. In Table 3, we can clearly see that our algorithm improves the segmentation performance while also achieving lower segmentation time compared to the two-dimensional OTSU algorithm. The improved genetic algorithm proposed in this paper optimizes the time complexity of the algorithm, greatly reducing the segmentation time while ensuring segmentation accuracy. Therefore, the algorithm proposed in this paper not only improves the quality of image segmentation, but also to a certain extent enhances the efficiency of the algorithm.

**Table 3:** Segmentation time of each algorithm (Unit: Millisecond).

Image	Iterative method	Bimodal method	One-dimensional OTSU	Two-dimensional OTSU	Algorithm in this paper
1	7.923	8.125	8.236	12.534	**10.241**
2	6.449	7.653	7.994	12.967	**11.692**
3	7.237	8.781	8.727	12.071	**11.734**
4	7.683	8.241	8.425	13.487	**11.528**

## 5 Conclusion

In this paper, we propose a two-dimensional OTSU inland ships multi-threshold image segmentation algorithm based on the improved genetic algorithm, which can enhance the accuracy and speed of image segmentation in complex navigable environments of inland rivers. This algorithm improves the crossover and mutation rates, and applies the improved genetic algorithm to the two-dimensional OTSU algorithm to solve the issue of algorithm time complexity. It optimizes the segmentation time of the OTSU algorithm while considering spatial location information, which helps avoid the algorithm from easily falling into local optimal solutions, and can handle relatively complex image segmentation problems of inland ships. The algorithm can significantly reduce image segmentation time, perform real-time ship image segmentation, and address the challenge of difficult acquisition of corresponding data sets for inland rivers, providing practical application value. Moreover, the algorithm can provide new ideas and methods for research in related fields and is also of reference significance for image segmentation problems under complex sea conditions.

In future perspectives, it is possible to consider using adaptive parameter adjustment to the structure of the genetic algorithm, such as adaptive evolution strategy, adaptive crossover probability, adaptive population size, and other aspects. In addition, it is also possible to consider applying it to image preprocessing under complex sea conditions. After multi-threshold image segmentation by this algorithm, further object recognition and detection can be performed.
